# Exercise and quality of life in patients with cystic fibrosis: A 12-week intervention study

**DOI:** 10.3109/09593985.2010.545102

**Published:** 2011-07-03

**Authors:** Anne Mette Schmidt, Ulla Jacobsen, Vibeke Bregnballe, Hanne Vebert Olesen, Thorsten Ingemann-Hansen, Mikael Thastum, Peter Oluf Schietz

**Affiliations:** 1 Department of Physiotherapy, Aarhus University Hospital, Skejby, Aarhus N, Denmark; 2 Department of Pediatrics, Aarhus University Hospital, Skejby, Aarhus N, Denmark; 3 Department of Sport Science, Aarhus University, Skejby, Aarhus C, Denmark; 4 Department of Psychology, Aarhus University, Skejby, Aarhus C, Denmark

## Abstract

It was hypothesised that increased exercise capacity is related to improved quality of life (QoL) in patients with cystic fibrosis (CF). A 12-week individually tailored unsupervised aerobic exercise programme was offered to 42 patients with CF. At the start and at the end of the exercise programme, data on QoL, current exercise habits and preferences, anthropometric data, exercise test, and lung function test were collected. Adherence was observed by a heart rate (HR) monitor. A total of 24 patients accepted to be enrolled in the exercise programme and 14 completed the programme. Another 14 patients declined to be enrolled in the exercise programme but completed the Cystic Fibrosis Questionnaire for Adolescents and Adults (CFQ-R 14+). Four patients did not want to participate at all. The 14 patients completing the exercise programme had a significantly increased VO_2max_, but they showed no significant change in total QoL score. However, the scores in the domain of treatment burden and emotional functioning increased significantly. There was no significant difference in QoL and lung function between patients participating in the exercise programme (*n* = 24) and non-participants (*n* = 14). A 12-week individually tailored unsupervised aerobic exercise programme where HR monitors were used significantly affected VO_2max_. Improvement in QoL could not be demonstrated in this study.

## INTRODUCTION

Cystic fibrosis (CF) is the most common hereditary disease in Caucasians, and no curative treatment has yet been found. In Denmark, life expectancy is now a median of 42 years (Ravilly et al, 2005).

Approximately 1 in 4,700 (Watters and Mehta, 2007) live births in Denmark is affected by the condition, and there are approximately 420 patients with CF in Denmark. CF primarily affects lung function and nutritional status (Klijn et al, 2003).

During the last decade, focus has been on exercise as an important element in the treatment of CF. Exercise has thus been incorporated in many standard treatment regimes, and exercise capacity is more often used as an outcome variable in intervention studies. Exercise capacity is currently used as an important parameter of disease status and progression (Orenstein and Higgins, 2005; Schneidermann-Walker et al, 2000; Turchetta, 2004), and it is well known that patients with a high exercise capacity have a longer life expectancy (Hebestreit et al, 2006; Klijn et al, 2004; Orenstein et al, 2004). Furthermore, it has been stated that aerobic capacity correlates with quality of life (QoL) measures, and changes in the former are associated with changes in the latter (Hebestreit et al, 2006).

Therefore, we designed a 12-week intervention study consisting of an individually tailored and unsupervised aerobic exercise programme to investigate the association between increased exercise capacity and changes in QoL in patients with CF. The exercise programme comprised everyday life activities adapted to the preferences of each individual patient. The patient was personally responsible for activating an HR monitor for every exercise session, indicating intensity, frequency, and duration and thus adherence to the programme.

It is our clinical experience that it can be difficult to motivate patients with CF to incorporate regular exercise into their everyday life. QoL is potentially negatively affected by the large burden of treatment and the complexity of the treatment evolving over years as CF progresses (Gee et al, 2005). It was our hope that an increase in exercise capacity and thereby an experienced increase in QoL could motivate the patients to adhere to the unsupervised exercise programme.

It was hypothesised that a 12-week individually tailored and unsupervised aerobic exercise programme in patients with CF could increase exercise capacity and thus QoL.

## MATERIALS AND METHODS

### Patients

Patients from the CF Centre at Aarhus University Hospital, Skejby, between 14 and 50 years of age, were invited. In our CF registry we found 42 eligible patients, and 24 accepted to participate in the study. A total of 14 of the 18 patients who declined participation filled in the Cystic Fibrosis Questionnaire for Adolescents and Adults (CFQ-R 14+).

Exclusion criteria were FEV_1_% <60, lung transplantation, debut of asthma or diabetes in the intervention period, and/or physical or psychological disability preventing participation.

All patients attended the CF Centre regularly, and during the study period the patients continued their regular therapy, including airway clearance therapy using a positive expiratory pressure mask, enzymes, vitamins, and other medical treatment.

Written informed consent was obtained from patients or parents. The study was approved by the Central Denmark Region Committees on Biomedical and Research Ethics, file number 20060052.

### Study design

The study period was 12 weeks. Measurements were performed at the start and at the end of the study. The pretests and posttests were identical.

### Measurements

Measurements were performed by the same assessors involved in the delivery of the intervention, and the patients were initially acquainted with the test procedures.

#### Quality of life

QoL was measured by using the Danish validated version (Bregnballe et al, 2008) of the disease-specific health-related QoL questionnaire, CFQ-R 14+ (Quittner et al, 2000). With a Chronbach's a ranging from 0.54 to 0.95, this seemed to be a reliable and valid instrument for measuring CF specific QoL (Bregnballe et al, 2008).

The CFQ-R 14+ consists of 49 self-reported items within 12 domains: 1) physical functioning (8 items); 2) vitality (4 items); 3) emotional functioning (5 items); 4) eating disturbances (3 items); 5) treatment burden (3 items); 6) general health perception (3 items); 7) social functioning (6 items); 8) body image (3 items); 9) role limitations (4 items); 10) weight problems (1 item); 11) respiratory symptoms (6 items); and 12) digestive symptoms (3 items). Answers were reported on a four-point scale rating frequency, difficulty, or truth, and selecting one of four statements that described the patient's situation. The scores ranged from 0 to 100 and higher scores equalled higher QoL.

#### Current exercise habits and preferences

To assess the patients' level of current physical activity, we asked them to fill in a short questionnaire (Wilhelmsen, Tibblin, Fordor, and Werkö, 1971). Furthermore, we interviewed the patients about their preferred physical activity.

#### Anthropometric data

Body weight was measured by using an electronic calibrated kilogram scale (BWB-800MA, Titan Corporation, Netherlands) and standing height using a stadiometer. The measurements were used to calculate the body mass index (BMI). For comparison between the different age groups, a BMI *z* score was calculated on the basis of Danish data on children and adults (Nysom, Mølgaard, Hutchings, and Michaelson, 2001).

#### Exercise test and maximum heart rate

Exercise capacity was measured as peak oxygen consumption rate (L/min). Before starting the incremental maximum exercise test, the patients warmed up on a voluntary level for 5 minutes on the cycle ergometer (Monark, Ergomedic 828E, Sweden). To reach the patients' maximum in 5-10 minutes, step-wise increments of 0.4 kilopond (kp) every 1.5 minutes were made, starting on individual levels from 0.8 to 1.8 kp, depending on the physical fitness of the patient. The patients were asked to choose a cadence between 60 and 85 revolutions per minute and to keep it during the test. The maximum level was reached when the patient could no longer maintain the cadence or to volitional fatigue. Patients were verbally encouraged throughout the test. The patients wore a close-fitting mask connected to an online respiratory gas exchange analyser (AMIS 2001, Innovision, Denmark) and computer system. Expired air was sampled continuously, and oxygen consumption and carbon dioxide production were measured every 10 seconds. VO_2max_ was determined as the peak oxygen uptake every 10 seconds throughout the test. VO_2max_ was normalised to body mass (VO_2max/kg_)- The HR was monitored (Polar, RS400, Finland) continuously every 5 seconds during the test, and the highest value was recorded. After the test, efforts were considered to be at a maximum level when three of the following four criteria were met: 1) total exhaustion; 2) HR above (220 - age) -10% 3) respiratory exchange ratio (the ratio between the measurements of VCO_2_ and VO_2_) above 1.11; and 4) the VO_2_ curve levelled off with a surge of less than 0.15 L/min from the last increment until the patient stopped the test.

#### Lung function

FEV_1_% was performed by using a Pneumotach spirometer (Masterscreen Body, Jäger, Germany). The pretest on lung function was performed on the day the patient entered the study, and the posttest on lung function was performed the day the patient ended the study.

### Intervention

To optimise adherence, each patient had the opportunity to select one or more physical activities for their individually tailored exercise programme. The patients chose different activities such as bicycling (including exercise bike), running, gymnastics, swimming, and dancing. Some patients performed more than one activity.

The patients were instructed to exercise at least half an hour three times a week for 12 weeks with an HR above 70% of their maximum HR calculated from the maximum HR of the pretest. An intensity of 70% of maximum HR has been used in several studies on patients with CF where an improvement in VO_2max_ was obtained (de Jong et al, 1994; Gulmans et al, 1999; Schneidermann-Walker et al, 2000; Selvadurai et al, 2002).

The exercise programme was intended to be a supplement to any exercise the patient already did.

Each training session was divided into two phases: 1) a warm-up phase of 5 minutes and 2) a training phase of at least 25 minutes. If the patients could not endure intensity above 70% of their HR maximum, they were allowed to stop the HR monitor and have a short break. HR was continuously monitored every 5 seconds by an HR monitor (Polar, RS400, Finland) to control training intensity. Exercise data were stored in the HR monitor as an exercise dairy and an evidence of adherence.

The patients were offered supervised training at the CF Centre once every second week, but no patients accepted this offer. We made motivational phone calls to all patients every week in the beginning of the exercise programme, and subsequent follow-up was planned individually. The motivational phone calls followed a nonstandardised semistructured interview guide. After the posttest exercise, data were transferred to a computer for analysis.

### Statistical analysis

Pretest values for different groups were analysed by a Mann-Whitney test. Dichotomous variables were tested by a chi-square test. Pretest and posttest values were compared by using a paired t test. A *p*-value of ≤ 0.05 was considered statistically significant. A Wilcoxin signed-ranks test was conducted to evaluate the impact of the exercise programme on CFQ-R14+ scores. By a Kruskal-Wallis test, CFQ-R 14+ scores of group 1 were compared with the CFQ-R 14+ scores of group 0 before starting the exercise programme and with group 2 (see classification of groups in Results).

## RESULTS

### Baseline data

The patients were classified as follows: group 0 = dropouts from the exercise programme (*n* = 10); group 1 = patients completing the exercise programme (*n* = 14); and group 2 = patients only filling in the CFQ-R 14+, non-participants (*n* = 14). Clinical characteristics of the 24 patients in the exercise programme (groups 0 + 1) are shown in Table 1.

**TABLE 1:** Clinical characteristics of the 24 patients entering the exercise programme (groups 0 + 1).

N	24
Gender M/F	15/9
Age range, years (median)	14–40 (19)
Genotype	
ΔF508/ΔF508	17 patients
ΔF508/other mutations	7 patients

Ten (group 0) of the 24 patients did not complete the exercise programme mainly due to practical reasons (e.g., lack of time and/or motivation and one patient had a knee injury at work). These 10 patients were not posttested. Fourteen (group 1) of the 24 patients were pretested, completed the exercise programme, and were posttested.

Engagement in vigorous activities in patients completing the exercise programme (group 1) did on average not meet the intended frequency and duration. Frequency was on average 2.31 times a week instead of three times a week, and duration was on average 79 minutes a week instead of 90 minutes a week. The patient exercising with the lowest intensity exercised with a 75% of maximum HR on average, and the patient exercising with the highest intensity exercised with an 86% of maximum HR on average (both patients, lowest and highest) exercising more than the recommended 70% of maximum HR. As shown in Table 2, we found no significant differences between group 0 and group 1 concerning physical baseline data.

**TABLE 2:** Comparison of physical baseline data between patients completing the exercise programme (group 1) and dropouts (group 0); median (25; 75% percentiles).

	Group 1 *n* = 14	Group 0 *n* = 10	*p*-value
FEV1%	89.4 (76.8;105.4)	92.8 (82.9;109.1)	0.447
BMI *z* score	−0.34 (−0.87;0.43)	0.01(−0.87;0.90)	0.520
VO_2max_ (L/min)	2.40 (1.93;2.78)	2.39 (2.18;3.12)	0.520
VO_2max/kg_ (mL/min/kg)	40.5 (34.5;45.8)	45.5 (36.8;47.0)	0.412
Age (years)	19 (15.5;27.0)	17 (15.5;19.5)	0.203
Gender	8M/6F	7M/3F	0.521

Fourteen (group 2) of 18 patients filled in the CFQ-R14+ but did not participate in the exercise programme. As shown in Table 3, no significant difference (*p* = 0.74) was found in FEV_1_% between participants in the exercise programme (groups 0 + 1) and non-participants (group 2). Similarly, there was no significant difference (*p* = 0.55) between participants completing the exercise programme (group 1) and non-participants (group 2).

**TABLE 3:** Comparison of baseline FEV_1_% between patients entering the exercise programme (groups 0 + 1), patients completing the exercise programme (group 1) and patients only filling in the CFQ-R 14+ (group 2).

	FEV_1_%; median (25:75%)
Group 0 + 1 *n* = 24	91.7 (79.7;106.3)
Group 1 *n* = 14	89.4 (76.8;105.4)
Group 2 *n* = 14	96.9 (79.6;104.1)

### Primary outcomes

#### Exercise capacity

**Figure 1:**
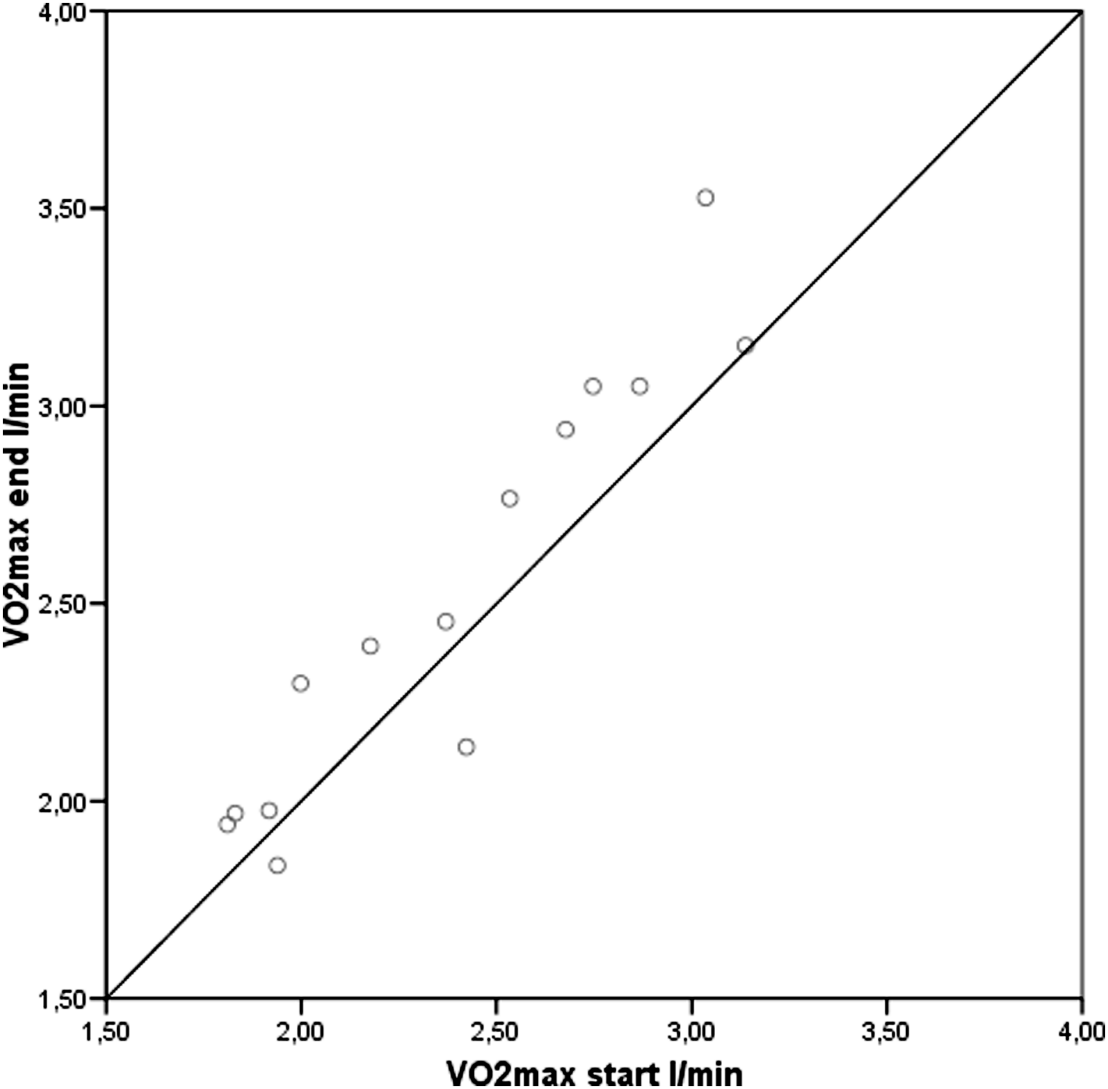
Patients above the diagonal improved their VO_2max_.

As seen in Table 4 and Figure 1, patients in group 1 significantly improved their VO_2max_, and their VO_2max/kg_ improved, though not significantly.

**TABLE 4:** Comparison of physical data of patients completing the exercise programme (group 1) before and after the intervention (median (25; 75% percentiles).

	Pretest	Posttest	*p*-value
FEV_1_% *n* =13 (*)	87.6 (74.8;105.8)	85.0 (76.5;99.0)	0.377
BMI *z* score *n* =14	−0.34 (−0.87;0.43)	−0.41(−0.92;0.49)	0.931
VO_2max_ (L/min) *n* =14	2.40 (1.93;2.78)	2.42 (1.97;3.05)	**0.014**
VO_2max/kg_ (mL/min/kg) *n* =14	40.5 (34.5;45.8)	42.5 (35.0;48.0)	0.057

* One patient did not complete the posttest on lung function because of cough.

#### Quality of life

In group 1 a significant increase was found in the domain of treatment burden from the scores before the intervention (M = 64.39; SD = 13.20) to the post-intervention scores (M = 76.19; SD = 8.56) *p* = 0.013; and in the domain of emotional functioning from the scores before the intervention (M = 81.43; SD = 17.77) to the postintervention scores (M = 86.19; SD= 13.70) *p* = 0.021 (Table 5). The eta-squared statistic (emotional functioning = 0.30; treatment burden = 0.50) indicated a large effect size. No significant differences were found in any other domains.

**TABLE 5:** Quality of life in patients completing the exercise programme (group 1) before and after the intervention.

Group 1 (*n* = 14)	Pretest (mean ± SD)	Posttest (mean ± SD)	*p*-value
Physical functioning	90.8 ± 14.5	93.8 ± 8.1	0.346
Role limitations	91.1 ± 12.0	92.9 ± 7.9	0.566
Vitality	59.5 ± 16.6	64.9 ± 11.4	0.204
Emotional functioning	81.4 ± 17.8	86.2 ± 13.7	**0.021**
Social functioning	74.6 ± 12.3	80.2 ± 13.6	0.191
Body image	83.3 ± 16.2	82.5 ± 14.9	0.783
Eating disturbances	94.4 ± 12.1	93.7 ± 15.5	0.655
Treatment burden	64.3 ± 13.2	76.2 ± 8.6	**0.013**
Health perceptions	66.7 ± 20.4	73.8 ± 17.2	0.091
Weight problems	83.3 ± 25.3	78.6 ± 24.8	0.317
Respiratory symptoms	77.8 ± 12.7	76.2 ± 13.5	0.371
Digestive symptoms	78.6 ± 19.7	80.2 ± 17.0	0.496

The CFQ-R 14+ scores of group 1 before the exercise programme were compared with the CFQ-R 14+ scores of group 0 (drop-outs) before the exercise programme and with the CFQ-R 14+ scores of group 2. No differences were found in any of the comparisons.

The internal consistency of each scale showed Cronbach's α coefficients ranging from 0.42 to 0.92 with coefficients <0.70 in the domains of role limitations (0.67); body image (0.62); social functioning (0.43); and digestive symptoms (0.42).

### Secondary outcomes

#### Anthropometric data

We found no significant differences in BMI *z* scores (Table 4).

#### Lung function

We found no significant differences in FEV_1_% (Table 4).

## DISCUSSION

To our knowledge, this is the first study in patients with CF investigating the response to an individually tailored and unsupervised aerobic exercise programme involving use of HR monitors. As far as we know, this is the first time when VO_2max_ and QoL measured by CFQ-R 14+ are primary outcomes. Our study also included QoL and FEV_1_% analysis of patients not participating in the exercise programme (group 2).

We did not find a significant difference in FEV_1_% between participants in the exercise programme (group 0 + 1) and non-participants (group 2). This may indicate that the patient's lung function did not seem to play any role in the willingness to participate in this study. Our individually tailored and unsupervised exercise programme had no positive or negative effect on FEV_1_%. The expected decline in lung function in patients with CF is likely to be between 2% and 3% annually. This suggests that studies shorter than 12 months may be unable to detect any difference in lung function (Orenstein and Higgins, 2005). Consequently, our study was not able to detect any effect on lung function.

We showed that patients with CF with a relatively good lung function (FEV_1_% above 60%) can increase their VO_2max_ significantly by participating in a 12-week individually tailored and unsupervised aerobic exercise programme. Our results are consistent with the results of other studies in both children and adults. Klijn et al (2004) found a significant change in VO_2max_ but not in VO_2max/kg_ following a 12-week anaerobic exercise programme in children with CF.

We did not find any significant increase in VO_2max/kg_. This is in contrast to several studies (de Jong et al, 1994; Gulmans et al, 1999; Hebestreit et al, 2010; Selvadurai et al, 2002; Turchetta et al, 2004). However, it is worth mentioning that these studies had different designs concerning type of intervention (e.g., aerobic exercise, anaerobic exercise, or a combination of these); the nature of the exercise (e.g., intensity, frequency, and duration); age of study population; duration of study; and outcome measurements.

Our study showed that 30 minutes of unsupervised exercise three times a week with an intensity of 70% of the maximum HR was enough to increase VO_2max_-Still, half of our patients did not manage to adhere to this exercise programme for an extended period of time. We expected that adherence in our study would be better than other studies on unsupervised exercise programmes because our patients could choose between different activities, knowing that adherence would never be as good as in supervised exercise studies. We still chose to have unsupervised exercise in our study to make it as realistic as possible. We believe that it is not realistic for logistical, financial, and geographical reasons that patients attend all exercise sessions supervised in the hospital. Our study underlines that HR monitors are preferred in unsupervised studies rather than exercise diaries because they are often poorly updated and seldom returned (Orenstein and Higgins, 2005). The HR monitors gave an impression of whether the patients exercised as instructed. Moreover, some of the patients told us that the HR monitor was a motivating factor. Apart from our study being unsupervised, the reason for the high dropout rate in our study may be the lack of emphasis on exercise as a part of routine CF treatment in Denmark.

Many participants complained about lack of time to do exercise but no one complained about the intensity. This is interesting in the light of our results on frequency, duration, and intensity. Engagement in vigorous activities in patients completing the exercise programme (group 1) did, on average, not meet the intended frequency and duration. However, the intensity of the lowest and the highest exercising patient was between 75% of maximum HR on average and 86% of maximum HR on average, both patients exercising with an intensity considerably higher than the recommended 70% of maximum HR. This suggested that the recommended intensity on 70% of maximum HR was not the reason for the high dropout level in our study. It can be debated whether the intensity was individualised. The intensity in relative terms was the same for all patients, whereas the absolute intensity was based on maximum HR of each patient. It may be that motivation, adherence, and QoL could increase as intensity increased and the duration of the exercise decreased.

The participation in our study was based on the patient's willingness, and we thus expected that all participating patients were motivated. This study did not allow patients to be randomised into a control group (no exercise) and participants were informed about this. At our CF Centre we encourage patients to gradually take responsibility of their disease when they become adolescents. We try to engage patients through continuous (educational) dialogue regarding treatment to enable the patient to take ownership of their treatment. Our belief is that we can achieve more by voluntariness of our patients and by their inner motivation. We had hoped that an increase in VO_2max_ would increase QoL and thereby the inner motivation to do exercise. A study by Arias Llorente, Bousono Garcia, and Dìaz Martin (2008) concluded that adherence has shown to improve with treatments that are perceived by patients as more important or influencing their QoL to a greater extent. Approximately 50% of people with chronic conditions do not adhere to treatment, and adherence reduces with the complexity of treatment. Previous studies in CF have found that adherence to chest physiotherapy and exercise is lower than other treatments (Myers, 2009).

Despite the proven benefits of exercise, the degree of physical activity generally undertaken in the CF population remains an issue of concern. The development of positive attitudes toward and a regular schedule for activity early in life may be important in determining an active style into adulthood (Prasad and Cerny, 2002). Earlier studies reported reasonable levels of compliance with exercise regimes. There are numerous possible reasons for the improved rates of adherence reported by these studies (e.g., the nature of activity, individualised programs, and whether activity is supervised). Programmes involving a single activity especially if prescribed over long periods of time are associated with poor long-term adherence (Prasad and Cerny, 2002).

Our patients were offered supervised training at the CF Centre once every second week but no patients accepted this offer. We made motivational phone calls to all patients every week in the beginning of the exercise programme and subsequent follow-up was planned individually. Nearly all the patients had one supervised exercise session during the intervention. Our strategy was to improve adherence by regular contact between us and the patient, not to supervise the programme as such but simply to show interest, enquire about the patient's well-being, and allow any queries to be answered. This method has been used previously as a part of an overall strategy to improve compliance in an exercise study by Schneidermann-Walker et al (2000). This study reported good rates of adherence.

We assumed that the short intervention period and individually tailored programme based on willingness to participate could lead to a relatively high adherence. However, several factors may influence adherence to any treatment regime, including a patient's willingness to participate in regular exercise over a period of time. These can be factors related to the disease process itself, gender, age, and external factors such as social support, perception of competency, self-esteem, enjoyment of activity, and factors associated with the patient's behaviour such as motivation and choice (Prasad and Cerny, 2002). The reason for the high dropout rate in this study could be the subject of further interest.

Our study failed to show that increased exercise capacity expressed as VO_2max_ could improve QoL. We found a statistically significant increase in scores of the domain of treatment burden and emotional functioning. The magnitude of the differences in the means were very large (eta-squared in treatment burden = 0.50; emotional functioning = 0.30). The increase in the scores of the domain of treatment burden was surprising. The explanation might be that the patients did not see the exercise program as a treatment burden, and the exercise program might have made them feel more energetic though this was not reflected in other domains such as vitality. Change of behaviour starts as a mental process, and this study indicated that changes did occur in patients who completed the exercise programme (group 1) by an improved emotional function score.

In this study the internal consistency of the CFQ-R 14+ scales measured by Chronbach's α failed to reach the recommended 0.7 in four of the scales. Three of these scales also showed a weak internal consistency in the validation of the CFQ-R +14; the fourth scale (role limitation) only showed a slightly lower Chronbach's α (0.67) than recommended.

The fact that the questionnaire failed to show an overall improvement in QoL might be explained by the complexity of the CFQ-R 14+. Retrospectively, we could not expect improvements in QoL after a 12-week intervention within the physical domains in CFQ-R 14+ such as eating disturbances and weight problems. Furthermore, group 1 had a high score in several of the physical domains in the pretest, leaving little room for improvement. The CFQ-R 14+ consists of 49 self-reported items within 12 domains, and the tool might have been too complex to show whether increased exercise capacity could result in increased QoL for patients with CF.

Two previous studies (Hebestreit et al, 2010; Klijn et al, 2004) on exercise have used CFQ to evaluate QoL, but not as a primary outcome. In contrast to our study, Klijn et al (2004) included anaerobic training. The intervention period was similar to ours, but this study was supervised. Klijn et al (2004) found a significantly higher score in the domain of physical function, but no change was found in other domains. Recently, in a partially supervised exercise programme for at least 6 months, Hebestreit et al (2010) did not find any significant effect on QoL except for the scale “Subjective Health Perception.”

Other intervention studies on exercise have measured QoL as a secondary outcome but with different measurements. In terms of degree of limitation in activities of daily living (ADL), de Jong et al (1994) found a decrease in the limitations in ADL. Using the “Quality of Well-Being Scale” Orenstein et al (2004) did not find any significant changes in QoL, but Selvadurai et al (2002) showed a significant difference in the aerobic exercise group. Earlier Orenstein, Nixon, Ross, and Kaplan (1989) assessed the “Quality of Well-Being Scale” and found a correlation between this scale and peak oxygen consumption. Gulmans et al (1999) found a significant improvement for the Total Competence Score as well as for the subscales “physical appearance” and “general self-worth” using the “Self Perception Profile for Children.” In summary, earlier studies have not provided a precise picture of the connection between exercise and QoL. Our study was not successful in fully clarifying this either.

There are some limitations of this study to be addressed. First, a 12-week study is a short time for a lifestyle change in patients with a chronic disease such as CF. Our aim was to increase exercise capacity and investigate if it had any positive effect on QoL. Bearing in mind that earlier studies have shown significant changes in either VO_2max_ or VO_2max/kg_ in 12 or less than 12 weeks of exercise (de Jong et al, 1994; Klijn et al, 2004; Selvadurai et al, 2002; Turchetta et al, 2004), we assessed that 12 weeks would be enough to increase VO_2max_ and thereby hopefully QoL. However, 12 weeks might not be enough to increase QoL even though it is enough to increase VO_2max_, and it could be interesting to study whether a longer study period would make a difference.

Second, our patients did not on average exercise at the recommended frequency and duration; instead, they exercised with a higher intensity than recommended. We do not know if the influence on QoL would have been different if our patients had exercised as recommended. Yet, the effect of the exercise programme was still in favor of the intervention and caused the expected increase in VO_2max_.

Third, in our exercise test we applied stepwise increments with fixed increment of 0.4 kp in determining the peak oxygen uptake. We are aware that some laboratories use continuous load increase to avoid one heavy load at one occasion. Stepwise increments could possibly cause some patients to finish the exercise test too early. However, in the present study all peak oxygen uptake measurements fulfilled three of four criteria (total exhaustion, HR above (220 - age) - 10%, respiratory exchanges ratio above 1.11, and/or the VO_2_ curve levelled off).

Fourth, the high dropout rate and thereby small sample size have weakened the conclusions. However, this has shown that the degree of encouragement and supervision in this programme may not have been sufficient and has to be individualised to achieve adherence to exercise as part of CF treatment.

Finally, due to lack of follow-up, we do not know if the patients in group 1 obtained a lifestyle change at the end of the study period. Hebestreit et al (2010) showed that at least 6 months of a partially supervised and individualised physical activity programme might be sufficient to cause persisting changes in activity behaviour and health. Whether our study is enough to induce this persisting change is unknown. A follow-up period in our study could have clarified this because a lasting effect after 12 weeks would be clinically relevant because such a programme can be realised relatively easily. Our aim was to measure the immediate effect of an unsupervised and individualised 12-week exercise programme on VO_2max_ and QoL, and our study was not designed as a follow-up study due to resources.

In conclusion, this study has shown that an effect on VO_2max_ can be achieved with an individually tailored and unsupervised aerobic exercise programme using HR monitors. A frequently postulated benefit of any physical exercise is an enhanced feeling of well-being. Thus, it was hypothesised that exercise capacity would be related to QoL in patients with CF. Improvement in QoL could not be demonstrated in this study.

Regular exercise is a very important factor in maintaining good health. This is even more important in patients with chronic diseases such as CF. Therefore, we still recommend that patients with CF increase or maintain as good a physical fitness as possible.
